# The role of photo-osmotic adaptation in semi-continuous culture and lipid particle release from *Dunaliella viridis*

**DOI:** 10.1007/s10811-014-0331-5

**Published:** 2014-05-13

**Authors:** Ryan W. Davis, Benjamin J. Carvalho, Howland D. T. Jones, Seema Singh

**Affiliations:** 1 Sandia National Laboratories, Livermore, CA 94551 USA; 2 Sandia National Laboratories, Albuquerque, NM 87185 USA; 3 Present Address: HyperImage Solutions, Rio Rancho, NM 87144 USA

**Keywords:** Microalgae, *Dunaliella viridis*, Algae bioproducts, Semi-continuous culture, Photo-osmotic adaptation, Lipid microparticles, Algal milking

## Abstract

Although great efforts have been made to elucidate the phenotypic responses of alga to varying levels of nutrients, osmotic environments, and photosynthetically active radiation intensities, the role of interactions among these variables is largely nebulous. Here, we describe a general method for establishing and maintaining semi-continuous cultures of the halophilic microalgal production strain, *Dunaliella viridis*, that is independent of variations in salinity and illumination intensity. Using this method, the cultures were evaluated to elucidate the overlapping roles of photosynthetic and osmotic adaptation on the accumulation and compositional variation of the biomass, photosynthetic productivity, and physiological biomarkers, as well as spectroscopic and morphological details at the single-cell level. Correlation matrices defining the relationships among the observables and based on variation of the illumination intensity and salinity were constructed for predicting bioproduct yields for varying culture conditions. Following maintenance of stable cultures for 6-week intervals, phenotypic responses to photo-osmotic drift were explored using a combination of single-cell hyperspectral fluorescence imaging and flow cytometry. In addition to morphological changes, release of lipid microparticles from the cells that is disproportionate to cell lysis was observed under hypotonic drift, indicating the existence of a reversible membrane permeation mechanism in *Dunaliella*. This phenomenon introduces the potential for low-cost strategies for recovering lipids and pigments from the microalgae by minimizing the requirement for energy intensive harvesting and dewatering of the biomass. The results should be applicable to outdoor culture, where seasonal changes resulting in variable solar flux and precipitation and evaporation rates are anticipated.

## Introduction

Species of the halophilic chlorophyte genus *Dunaliella* are amongst the most commercially relevant microalgae worldwide. *Dunaliella* spp. have been explored for industrial scale production in outdoor cultivation facilities for a variety of value-added and antioxidant compounds used in the neutraceutical, cosmetic, and biochemical industries (Borowitzka 1986). In particular, this genus serves as a potential biological source of food dyes, vitamin A, tocopherols, glycerol, lutein, peptides, and beta-carotene (Ben-Amotz and Avron 1982; Jin and Melis 2003; Lewin 1992). Furthermore, *Dunaliella* and related species have lipid and fatty acid compositions that are suitable for biodiesel production (Azachi et al. 2002; McCormick et al. 2006; Peeler et al. 1989; Tornabene et al. 1980), although the potential for exploiting this feedstock for biofuels application has been only marginally investigated. A recent assessment of biofuel production (i.e., bioethanol and biodiesel) from microalgae suggests that the optimal composition of the biomass should approach 60 % lipids, 30 % starch, and 10 % protein (Martin and Grossmann 2013); however, this is rarely achieved in high productivity outdoor cultures. In an integrated production model, extraction of the co-products present in the biomass will likely continue to play a large role for the economic viability of producing the feedstock (Wijffels et al. 2010). Furthermore, recent advances in genetic manipulation of *Dunaliella* for expression of recombinant therapeutics could significantly expand the prospects for molecular farming in this species (Barzegari et al. 2010; Polle and Song 2009).

In addition to the potentially lucrative biomaterials produced by *Dunaliella*, the robust growth in high salinity environments is biotechnologically and agriculturally desirable for development of non-arable land resources—a key sustainability concern for production scale-up (Borowitzka and Moheimani 2013; Pate et al. 2011). Whereas high salinity is inhibitory to photosynthesis in most higher plants, high salinity has been found to enhance photosynthetic activity and carbon assimilation in *Dunaliella* (Booth and Beardall 1991; Liska et al. 2004). Other adaptations contributing to halotolerant osmoregulation in *Dunaliella* include the replacement of a rigid cell wall with a flexible mucin layer (Kirst 1989), production of glycerol as an intracellular compatible solute (Ben-Amotz and Avron 1973; Borowitzka and Brown 1974; Chitlaru and Pick 1991), enhanced synthesis of carotenoids as a protectant against photo-oxidative stress (Jin and Melis 2003; Zamir et al. 2004), and the production of extracellular halotolerant enzymes that retain structural stability and solubility over a wide range of salt concentrations (Booth and Beardall 1991; Premkumar et al. 2005). Thus, the prospects for combining high biomaterials productivity with the ability to grow in extreme saline environments make *Dunaliella* a genus of continued interest for commodity-scale bioresource development.

Coupling effective means of harvesting and product isolation to high biomass yields has been identified as a major requirement for large scale microalgae production (Greenwell et al. 2010). Semi-continuous culture of microalgae provides the opportunity to optimize biomass yields by overcoming growth lags by minimizing the inhibitory effects of light shading in high-density cultures and slow onset in low-density inocula. Furthermore, semi-continuous culture systems minimize land footprint, favor the production of high growth-rate strains, and enable standardization of the resultant biomass composition. A potential complimentary approach to semi-continuous culture involves non-lethal isolation of specific analytes of interest (esp. lipids and carotenoids) from the active culture, sometimes referred to as algal milking. Although non-lethal milking of hydrocarbons has been demonstrated for *Botryococcus braunii* (Moheimani et al. 2014), strategies intending to milk bioproducts from *Dunaliella* (Hejazi et al. 2004; Kleinegris et al. 2010a) have in fact resulted on lysis of the cells from exposure to organic solvents (Kleinegris et al. 2011). In this manuscript, we detail observations which indicate that semi-continuous cultures under osmotic drift can provide disproportionally high release of lipid microparticles from the algae cells compared to cell lysis. This finding indicates the potential for reversible osmotic permeation of *Dunaliella viridis* for release of lipid phase bioproducts.

In order to identify the dominant factors impacting the return on investment from biomass production, accurate measurement of a wide range of physiological parameters over a multiplicity of controlled inputs is required. In recent years, a variety of spectroscopic and imaging methods have been employed for quantifying total biomass, metabolic biomarkers, lipid speciation and content, and high-value algal biomaterials, such as carotenoids (Davis et al. 2012; Kleinegris et al. 2010b; Vermaas et al. 2008; Wu et al. 2011). In this study, we employed the combination of established and recently developed spectroscopic techniques, including hyperspectral confocal fluorescence imaging, excitation modulated fluoromety, and flow cytometry in conjunction with common fluorescent and colorimetric indicators to track the morphological, metabolic, and biomaterials parameters of *D. viridis* across a variety of photo-osmotic conditions. Together, these data should be broadly applicable for identifying biomass yield and composition for maximizing return on algae biomass production.

## Materials and methods

### Algal cultures

*Dunaliella viridis* was cultured from UTEX LB200 stock (Borowitzka and Siva 2007) in enriched artificial seawater medium (Berges et al. 2001) supplemented with 5 % *v*/*v* sterile-filtered soil seawater (UTEX, USA), and additional nitrate (3 mM total) and phosphate (66 μM total). Three distinct salinity levels were maintained: “low salinity” (LS) which corresponds to seawater, “moderate salinity” (MS) which corresponds to seawater with the addition of 1 M NaCl, and “high salinity” (HS) which corresponds to seawater with the addition of 2 M NaCl. Modified ESAW was prepared as per the recipe in Table 1.

**Table 1:** Compositional details of the semi-continuous *D. viridis* cultivation medium. All quantities, except for the varying NaCl component (Low Salt: LS, Medium Salt: MS, and High Salt: HS), were identical for each photo-osmotic growth condition

Medium component	Quantity
Artificial seawater	
Low Salt (LS): NaCl	362.7 mM
Medium Salt (MS): NaCl	1.3627 M
High Satl (HS): NaCl	2.3627 M
Na_2_SO_4_	25.00 mM
KCl	8.030 mM
NaHCO_3_	2.067 mM
KBr	0.725 mM
H_3_BO_3_	0.433 mM
NaF	0.0657 mM
MgCl_2_·6H_2_O	47.18 mM
CaCl_2_·2H_2_O	9.134 mM
SrCl_2_·6H_2_O	0.0214 mM
Nutrients	
NaNO_3_	3.00 mM
Na_2_HPO_3_	0.066 mM
Trace metals	
Na_2_EDTA·2H_2_O	14.86 μM
FeCl_3_·6H_2_O	6.562 μM
MnSO_4_·4H_2_O	2.42 μM
ZnSO_4_	0.245 μM
CoSO_4_	56.9 nM
Na_2_MoO_4_·2H_2_O	0.520 μM
Na_2_SeO_3_	1.0 nM
Vitamins	
Thiamine	0.297 mM
Vitamin B12	1.47 μM
Biotin	4.09 μM
Soil seawater	5 % *v*/*v*

### Algal culture

Culturing was performed in 500 mL cylindrical flasks (200 mL headspace volume) in a temperature regulated (27 °C) chamber equipped with a custom Peltier-cooled variable intensity LED strip illuminator system (6,500 K solar spectrum) and humidified air-injected bubbling (200 cm^3^ min^−1^). This system supports simultaneous implementation of three independent photosynthetically active radiation (PAR) levels. Culture flasks were placed directly on the illuminator and the intensities in the samples were adjusted to 50 μmol photons m^−2^ sec^−1^ for “low” PAR (LL) and 275 μmol photons m^−2^ sec^−1^ for “high” PAR (HL) measured with a US-SQS/L spherical micro- quantum sensor (Walz, Germany) positioned 2.54 cm from the illumination source in media-filled flasks.

### Hyperspectral confocal fluorescence imaging

Hyperspectral confocal fluorescence imaging was employed to facilitate morphological variation and identification and assignment of the dominant spectral components present in fluorescence images and flow cytometry data. For imaging of *D. viridis*, cells were collected, dark adapted, and delivered to a glass slide and coverslip assembly. Hyperspectral fluorescence images were collected using a 60x 1.4NA microscope objective with scanned 488 nm laser excitation and 512 EMCCD detector channels, corresponding to each pixel in the image having a spectral emission range of 500–800 nm as described previously (Sinclair et al. 2006). In order to obtain accurate spectral estimates of the non-chlorophyll-related spectral components (in the 500–625 nm spectral region), two sets of image data were acquired per field, first using an absorptive filter (Newport BG-40) that attenuates the chlorophyll signal and ∼150 μW laser excitation, and a second without the filter and ∼0.5 μW laser excitation. This method is necessary to remove the relatively large Poissonian noise contribution from the high level of chlorophyll fluorescence on the lower intensity pigment emissions for downstream analysis. In addition to fluorescence images of single algae cells, dark images were also collected to account for instrument noise (Jones et al. 2008). The resulting spectral image data were then subject to multivariate curve resolution (MCR) analysis using imageMCR, an in-house software package written in Matlab (Mathworks, Inc.). The imageMCR software implementation uses a constrained iterative alternating least squares algorithm (Haaland et al. 2009) to discover and model the spectral shapes and corresponding relative intensities of spectral components contained within the images. For the MCR analysis, image data were combined across all of the photo-osmotic culture conditions and analyzed as a batch to obtain a common set of pure component spectra. Spectral assignments were made and confirmed by comparison to literature spectra. Cell diameter estimation was obtained from single-cell fluorescence imaging data based on pixel size calibration via translation of 100 nm dia. fluorescent microspheres.

### Biomass estimation

Semi-continuous cultures corresponding to the various PAR intensities and salinity levels (later referred to by photo-osmotic combinations, e.g., HL×HS corresponds to high PAR × high salinity, LL×LS to low PAR × low salinity, etc.) were maintained for three 6-week intervals in mid- to late log-phase by harvesting the volumetric growth as indicated by the near IR attenuation of the culture (800 nm optical density) at 48 h increments. The spectrophotometric method was calibrated to account for the potential for significant differences in the attenuation profile of the microalgae arising from differences in the refractive indices of the varying salinity media and the cells (based on compositional variation) and impacts of cell size variation on the scattering and absorption coefficients. This was achieved by performing serial dilution on the initial biomass samples and analyzing each using hemocytometry, flow cytometry, and VIS-NIR spectrophotometry to generate linear regression-based estimates of cell count from spectrophotometric data generated at each harvesting increment. Following each harvesting increment, the removed volumes were replaced with equal volumes of fresh medium. Multifactorial data collection was initiated at the beginning of the fourth harvesting increment to provide results consistent with adapted stable cultures, and continued for the 6-week interval, corresponding to 20 samples per condition. Following the 6-week interval, the semi-continuous culture was continued for an additional week, however the replaced media consisted of one of the osmotic extrema (e.g. LS medium was added to HS cultures) to investigate the impacts of hypo- and hypertonic drift.

For each of the assays described below, samples for analysis were collected coincident with harvest, prior to supplementation with fresh media. Live cell assays, including hyperspectral fluorescence imaging, flow cytometry, excitation modulation fluorescence, and the electrode-based measurements were performed immediately following sample collection; samples for algae biomass yield (as ash-free dry weight, AFDW) and composition assays were centrifuged (750×*g*, 5 min), decanted, lyophilized, and aggregately stored at −80 °C for later subsequent analysis. Final biomass quantity was assessed by gravimetric determination of AFDW according to established methods (Zhu and Lee 1997). Estimation of relative cellular motility was obtained by comparing cell counts (using flow cytometry) from 100 μL cell aliquots collected 5 mm below the surface of the medium before and following a 10 min (unstirred and non-illuminated) resting period following sample collection.

### Biomass composition: protein content

Extraction of proteins was performed by resuspending 5 mg of washed and lyophilized *D. viridis* biomass in 5 mL of 100 mM phosphate-buffered saline (PBS, pH 6.5) and subjecting the suspension to probe ultra-sonication (550 Sonic Dismembranator, Fisher Scientific) on ice at the microtip limit on 50 % duty cycle for 3 min, followed by gentle agitation at 4 °C for 16 h. Following centrifugation (7,500×*g*, 15 min, 4 ° C), the quantity of soluble proteins were determined from the supernatant using the bicinchoninic acid method (BCA) with bovine serum albumin as a colorimetric standard. Insoluble proteins were then extracted from the remaining centrifugation pellet by resuspension in 5 mL of 5 % (*w*/*v*) trichloroacetic acid (TCA), followed by bath sonication at 90 °C for 3 h. The resuspended material was then centrifuged (10,000×*g*, 10 min, 4 °C) and the supernatant collected for subsequent determination of insoluble carbohydrate, while the pellet was resuspended in 3 mL 1 N NaOH, and stored for 16 h at 4 °C. Following solubilization in base, the biomass fraction was neutralized with 3 mL 1 N HCl, and centrifuged. The fraction of insoluble proteins was then determined from the supernatant using the BCA method as described previously.

### Biomass composition: carbohydrate content

Determination of soluble and insoluble carbohydrates was performed using the colorimetric phenol-sulfuric acid method using a d-glucose standard (Kochert 1991). Prior to analysis, proteins were removed from the solubilized biomass by precipitation using TCA and sodium deoxycholate (DOC) (Clayton et al. 1988). Specifically, 400 μL of 1.4 M TCA and 50 μL of 9 mM DOC were added to 5 mL of the PBS supernatant (in the previous section Biomass composition: protein content), incubated at room temperature for 10 min and subsequently centrifuged (7,500×*g*, 10 min, 4 °C). Insoluble carbohydrates were extracted from the supernatant after removal of the structural proteins (described above), according to established protocols (Bird 1982). For quantification of both the insoluble and soluble carbohydrate fractions, 250 μL of the samples were combined with 500 μL of 4 % aqueous phenol and 1.25 mL of 12 N H_2_SO_4_, incubated for 10 min in the dark and the absorbance was measured at 487 nm. No indications of false-positives for carbohydrate were observed by the presence of glycerol in control samples.

### Biomass composition: total lipid content

Lipids, including triacylglycerol, polar (membrane) lipids, and pigments were extracted according to Folch et al. (1957). Specifically, 10 mL of 2:1 (*v*/*v*) chloroform-methanol was added to 200 mg of lyophilized biomass and placed on a shaker table (240 rpm) for 15 min. The lipid phase was then separated from the extracted biomass in a funnel (retaining any persistent emulsion) and dried to completion under a stream of dry N_2_ followed by vacuum desiccation (16 h, 30 ºC). The total lipid content was then determined gravimetrically. For mass balance considerations, the quantities of chlorophylls and carotenoids (discussed below) were subtracted from the total lipid content for each sample.

### Biomass composition: chlorophyll and carotenoid content

Determination of the chlorophyll and carotenoid pigments content was performed spectrophotometrically following methanol extraction, using consistent equations (Ritchie 2006, 2008) for chlorophyll and carotenoids (Lichtenthaler and Wellburn 1983), respectively. Specifically, 3 mL analytical grade methanol (Sigma) was added to 10 mg lyophilized biomass in a shatter-proof and sealable container. The samples were then heated to 60 °C and briefly vortex mixed, followed by six cycles of freeze-thaw. Freeze-thaw cycles were performed by alternating between liquid N_2_ and 60 °C sonication baths upon visual inspection of solid–liquid phase change behavior, with brief vortex mixing between each transition. The cell suspensions were then centrifuged (7,500×*g*, 4 min, room temperature) and the supernatant retained for UV–VIS absorbance measurements. Absorbance values measured at 665 and 470 nm were subsequently used to determine the concentrations of chlorophyll and carotenoid.

### Biomass composition: glycerol content

Determination of glycerol content was according to methods established for *Dunaliella* (Chitlaru and Pick 1989) using a glycerol standard. Specifically, 2 mL of periodate reagent (65 mg NaIO_4_, 10 mL glacial acetic acid, 7.7 g ammonium acetate), and 5 mL of acetylacetone reagent (2.5 mL acetylacetone, 247.5 mL isopropanol) were added to 2.5 mg of lyophilized biomass and agitated for 30 min at 45 ºC. After cooling to room temperature, the absorbance of the sample was recorded at 410 nm.

### Culture pH and nitrate consumption

The bulk pH and nitrate concentrations of each of the cultures were measured at each of the sample intervals for integration with the data matrix. pH was measured using a standard calibrated pH/conductivity meter (Orion, USA). Nitrate concentration was measured using a DJM Micro Nitrate ISE (Lazar Research Laboratories, Inc., USA) in conjunction with a double junction reference electrode filled with a 0.1 M lithium acetate solution. An ionic strength adjuster (ISA) was employed at 1:1 volume ratio with the freshly collected algae sample to minimize interference from chloride ion in the media. The ISA was comprised of 60.3 mM K_2_SO_4_ (Sigma) and 10 mM Ag_2_SO_4_ (Sigma), and adjusted to pH 2.75 with 0.1 M H_2_SO_4_. Standard curves for nitrate were generated for each of the salinity adjusted media using media controls spanning a range of NaNO_3_ from 0–3 mM. Quantitation of nitrate uptake (Δ[NO_3_^−^]) was performed by comparing readings made immediately following re-addition of medium and at the subsequent harvest interval.

### Flow cytometry

Flow cytometric assays, including assessment of whole cell vs. debris content, cell size, cellular granularity, relative chlorophyll *a*/*b* content, relative carotenoid content, relative TAG content by Nile Red (9-(diethylamino)-5*H*-benzo[*a*]phenoxazin-5-one) (Cooksey et al. 1987), intracellular calcium content by Fura-Red AM (glycine, *N*-[2-[(acetyloxy)methoxy]-2-oxoethyl]-*N*-[5-[2-[2-[bis[2-[(acetyloxy)methoxy]-2-oxoethyl]amino]-5-methylphenoxy]ethoxy]-2-[(5-oxo-2-thioxo-4-imidazolidinylidene)methyl]-6-benzofuranyl]-, (acetyloxy)methyl ester) (Novak and Rabinovitch 1994), and intracellular pH by SNARF-1 (carboxy-seminaphthorhodafluor) (Wieder et al. 1993) were performed using an Accuri C6 Flow Cytometer (BD Biosciences). Measurements were performed using sheath fluids with a range of NaCl from 0 to 2.5 M. Calibration of the forward scatter (FSC) detector channel was performed using the following sizes of fluorescent polystyrene beads (FluoSpheres, Invitrogen, USA) suspended in double distilled filtered (20 nm, Whatman, USA) water: 2.0, 7.52, 9.7, and 15.41 μm. Calibration of the fluorescence detector channels was performed using constant diameter (3 μm), 8-intensity level fluorescent validation beads (Spherotech, BD Bioscience). Following dark adaptation, staining, and pipette stirring, measurements were performed at a flow velocity of 35 μL min^−1^, corresponding to a core flow diameter of 16 μm, with instrumental gating imposed at forward scatter values of <100 (i.e., below the nominal detection limit of the instrument). Analysis of the data was performed using software provided by Accuri or by the FloJo software package (Tree Star, Inc.). Particle diameters were determined from FSC intensity and comparison to signals obtained by the polystyrene beads. Relative cellular granularity was determined by from the side scatter (SSC) intensity. Relative chlorophyll *b* content was determined by the FL3 fluorescence intensity (excitation wavelength, 488 nm; emission filter, >670 nm) and relative chlorophyll *a* content was determine by the FL4 fluorescence intensity (excitation wavelength, 640 nm; emission filter, 675 ± 12.5 nm). Relative carotenoid content was determined by the FL2 fluorescence intensity (excitation wavelength, 488 nm; emission filter, 585 ± 20 nm). Estimation of cellular debris in the medium relative to intact cells was obtained from 2D scatter plots of FSC and chlorophyll a (FL4) intensity, with particles not belonging to the dominant cell population counted as debris. This debris count was corrected by subtracting average counts in the non-cell region from data collected using sterile filtered medium appropriate for the specified culture condition for the same amount of time that data was collected on the algal populations (1 minute). Nile Red staining was performed by adding 5 μL mL^−1^ sample of 5 μL mL^−1^ as an acetone bolus 10 min prior to the beginning of a measurement. Nile Red fluorescence intensities were recorded in the FL2 detector channel and corrected by subtracting fluorescence detected in that channel from unstained cells. Fura-Red staining was performed by solubilizing 50 μg the acetomethoxy ester (Fura-Red AM, Life Technologies) in 20 μL of reagent grade DMSO and subsequently combining this dye solution with 980 μL of a 10 % (*w*/*v*) 0.2 μm filtered aqueous Pluronic-127 solution at 40 ºC, followed by intermittent vortexing for ∼1 min. Fifty microliters of the resulting fluorescent stain was then added to 225 μL of algae cell suspension (final [Fura-Red] = 10 μM) and incubated for 30 min at 27 °C prior to analysis. Fura-Red fluorescence intensities were recorded in the FL1 detector channel (excitation wavelength, 488 nm; emission filter, 510 ± 15 nm) and corrected by subtracting fluorescence detected in that channel from unstained cells. SNARF-1 staining was performed by solubilizing 50 μg the acetomethoxy ester (SNARF-1AM, Life Technologies) in 20 μL of reagent-grade DMSO and subsequently combining this dye solution with 980 μL of a 10 % (*w*/*v*) 0.2 μm-filtered aqueous Pluronic-127 solution at 40 ºC, followed by intermittent vortexing for ∼1 min. Twenty-five microliters of the resulting fluorescent stain was then added to 225 μL of algae cell suspension (final [SNARF-1] = 10 μM) and incubated for 30 min at 27 °C prior to analysis. SNARF-1 ratiometric fluorescence intensities were recorded in the FL2 and FL3 detector channels, and corrected by subtracting the average fluorescence intensity detected in these channels from unstained cells. Final pH values from the SNARF-1 ratiometric fluorescence intensities were obtained by comparison to literature values.

### Excitation modulated fluorescence

Estimation of photosystem parameters was performed as described previously according to a Stern-Volmer process (Kramer et al. 2004; Schreiber 2004).

The maximum PSII quantum yield [*Y* ^max^(II)] is obtained from the equation,

$$ {Y}^{\max }(II)=\left({F}_m-{F}_0\right)/{F}_m. $$

Following the initial PAR exposure, the subsequent elevated photon flux provides the effective PSII quantum yield, *Y*(II), obtained by the previous expression, with *F*_m_ = *F*_m_′, i.e., the modified *F*_m_ obtained at the fluorescence plateau during the second saturation pulse, and *F*_0_ = *F*, i.e., the modified *F*_0_ obtained under increased, physiologically relevant PAR intensities. The non-photochemical quenching parameter, NPQ, is calculated according to a diffusion-limited Stern-Volmer quenching mechanism using the aforementioned fluorescence parameters according to the equation,

$$ \mathrm{NPQ}=\left({F}_{\mathrm{m}}-{ F\mathit{\hbox{'}}}_{\mathrm{m}}\right)/{ F\mathit{\hbox{'}}}_{\mathrm{m}}. $$

The effective PSII quantum yield, *Y*(II), sums to unity with the regulated, *Y*(NPQ), and unregulated, *Y*(NO), energy dissipation mechanisms to account for the PAR absorption in PSII. The coefficient of photochemical quenching, *qL*, required for differentiation of the two energy dissipation mechanisms is obtained following exposure to the far red illumination to obtain the minimal fluorescence yield, *F*_0_′, and the aforementioned effective PSII quantum yield parameters according to the equation,

$$ qL={ F\mathit{\hbox{'}}}_0\left({ F\mathit{\hbox{'}}}_{\mathrm{m}}-F\right)/\left({ F F\mathit{\hbox{'}}}_{\mathrm{m}}-{ F F\mathit{\hbox{'}}}_0\right). $$

Finally, *Y*(NO) and *Y*(NPQ) are calculated using the following equation, and by incorporating the unity constrain among these parameters and *Y*(II),

$$ Y\left(\mathrm{NO}\right)={\left(1+\mathrm{NPQ}+ qL\left({F}_{\mathrm{m}}/{F}_0-1\right)\right)}^{-1}. $$

The aforementioned photosynthetic parameters and photosynthetically induced NAD(P)^+^/NAD(P)H redox turnover [ΔNAD(P)H] were measured using a Pulse Amplitude Modulated (PAM) fluorimeter (Dual PAM-100, Walz, Germany). In the present study, data was collected using the rapid light curve (or RLC) routine (White and Critchley 1999). Chlorophyll fluorescence (700 ± 20 nm) was detected in response to the measuring light and ∼1 ms duration saturation pulses applied following exposure to stepped increases of actinic excitation intensity (635 nm, stepping increments = 50, 100, 150, 275, 450, 650, 1050, 1,600 μmol photons m^−2^ sec^−1^) over ∼5 minutes, immediately followed by far red illumination for 30 sec (760 nm). Measurement of NAD(P)H flux was performed using a similar routine, however, NAD(P)H fluorescence was excited at 365 nm and detected at 485 ± 65 nm during the course of the RLS scan. Prior to all measurements, the algae suspensions were dark adapted for 15 min and briefly pipette stirred.

## Results

**Fig. 1:**
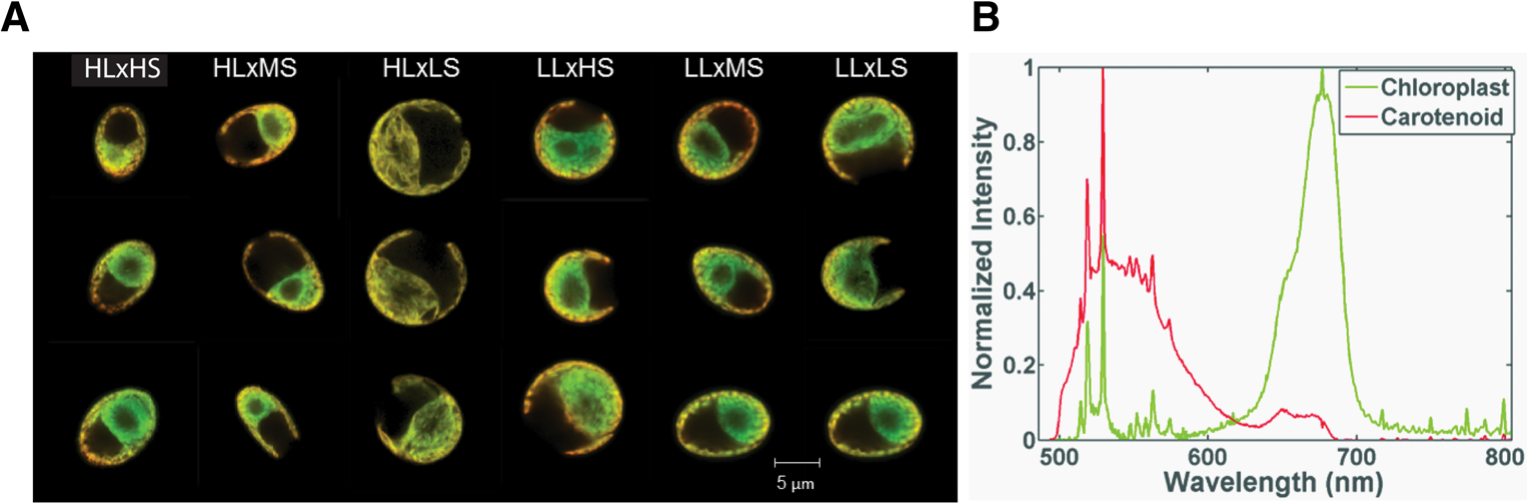
Hyperspectral confocal fluorescence images of *D. viridis* adapted to a variety of photo-osmotic conditions. **a** Representative hemispherical sections of individual *D. viridis* cells depicting phenotypic variations dictated by the various salinity and PAR illumination regimes. **b** Fluorescence emission components obtained by MCR analysis and used for intensity mapping of the cells depicted in **a**

*Dunaliella viridis* cells cultured in the various photo-osmotic conditions were characterized using confocal fluorescence hyperspectral imaging to couple the compositional variation and photosynthetic and biochemical indicators with spectroscopic signatures and spatial data, including cell size and morphology. Figure 1 provides representative images (a) and a corresponding set of spectrally unmixed fluorescence components (b) obtained from batch processing of the aggregated imaging data using MCR.

The intensity images in Fig. 1a were generated according to the relative abundance of the spectral components shown in Fig. 1b. Two distinct spectral features dominate the emission spectrum for all of the *D. viridis* imaging data, denoted as “chloroplast” and “carotenoid” in Fig. 1b. The chloroplast spectral component (green) is characterized by a high intensity Chl *a* photosystem emission (peak at ∼680 nm) colocalized with low levels of carotenoid emission; the carotenoid spectral component (red) is characterized by a minor, but broad chlorophyll fluorescence (∼660–680 nm) with high levels of carotenoid emission (∼520–600 nm). The sharp peaks within these spectra are not due to noise; instead they are a result of resonance enhanced Raman scattering. Typically, Raman bands would not be observed in a fluorescence measurement; however, resonance enhancement arising from absorption of the excitation laser near the electronic transition of the carotenoids is sufficient to amplify a subset of Raman-active modes by several orders of magnitude (Robert 1999). Specifically, these bands were assigned to three major vibrational modes of carotenoids, the C═C stretch (1,529 ± 10 cm^−1^ or 527 nm), the C–C stretch (1,157 ± 10 cm^−1^ or 519 nm), and a C–C bend (1,004 ± 10 cm^−1^ or 514 nm). Although progressive shifts in the wavenumber of the C═C stretching band are correlated to increased polyene chain length, deviations in the exact frequency shift should be expected by interactions with the host matrix (de Oliveira et al. 2010). In addition to providing indication of the relative abundance and cellular localization of these spectral components, the data also provide an estimate of cell size and granularity for subsequent data interpretation.

**Fig. 2:**
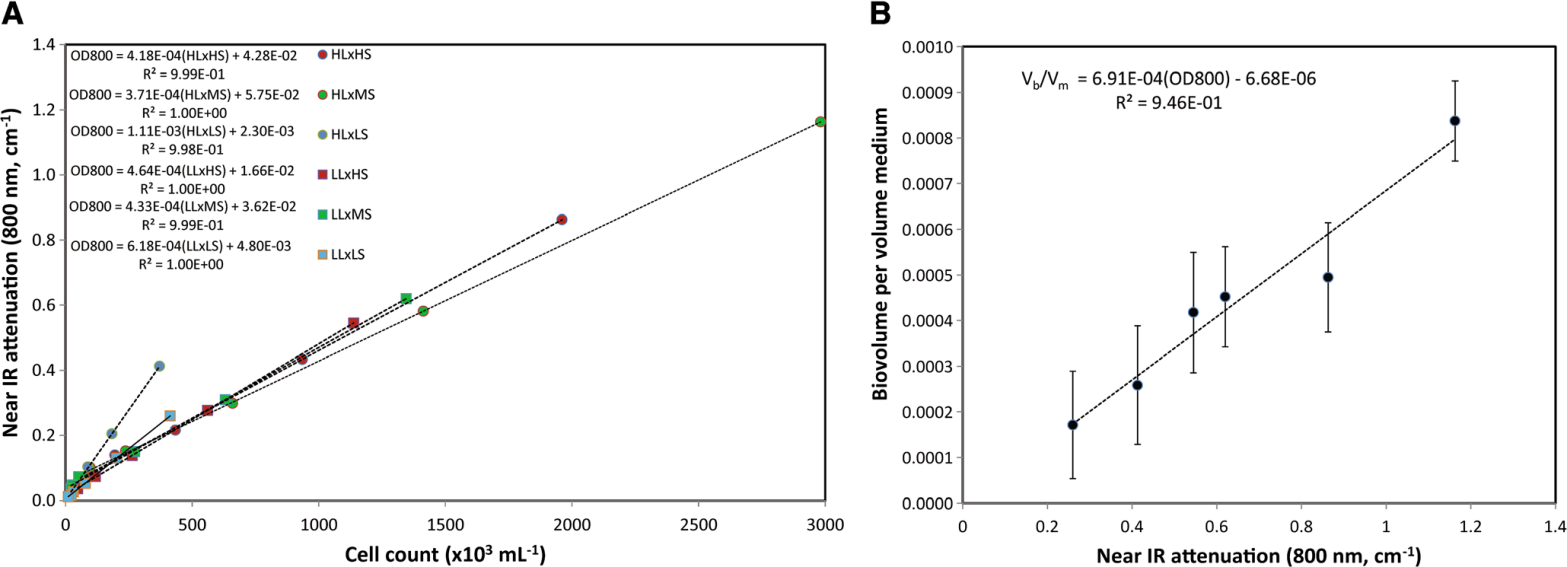
Near IR estimation of *D. viridis* biomass of varying composition and morphology cultured in varying photo-osmotic conditions. **a** Near IR (800 nm) attenuation of serially diluted log-phase cultures as a function of cell counts measured by flow cytometry. **b** Hydrated biomass volume calculated by number of cells and the effective cell volume obtained from confocal fluorescence imaging

Semi-continuous harvesting of the *D. viridis* biomass at log phase was achieved using a calibration data set collected from initial cultures representing each condition in the photo-osmotic adaptation matrix. Combining cell counts with the effective cell diameters obtained from fluorescence imaging data allowed prediction of the total biomass from the bulk spectrophotometric data (see Fig. 2). Correlation of the hemocytometry and flow cytometry data yielded nearly identical total cell counts (data not shown), however the standard deviation values of the hemocytometry data was significantly higher, therefore flow cytometry was chosen as the method of choice for linear regression analysis with VIS-NIR. Figure 2a depicts the correlation between the near IR attenuation and cell counts obtained from flow cytometry for each of the serially diluted cultures. For all conditions tested *R* ^2^ > 0.99 were obtained, however, significant differences were observed in the slopes of the various culture conditions, indicating significant differences in the optical attenuation profile of the cells. Examination of the extrema of the near IR attenuation per cell (viz. HL×MS and HL×LS) suggest that the cell count predicted by the 800 nm optical densities varies by up to ∼300 % based on physiological changes introduced by the combination of salinity and PAR intensity investigated here. This deviation in the near IR attenuation per cell is nearly identical to the ratios of cell volumes observed from the confocal microscopy studies. In Fig. 2b, the volume of the native (hydrated) biomass was calculated by multiplying the cell counts with the average cell volumes and correlated to the near IR attenuation for the undiluted log-phase cultures. In this case, regression analysis indicates a linear trend for an increasing volume fraction of the biomass with increasing 800 nm optical density (*R* ^2^ = 0.946). Together, these data suggest that despite substantial differences in cell size and biochemical composition, near IR attenuation is nonetheless a consistent indicator for total biomass accumulation. A primary implication of this finding is that water content in the cells is largely invariant despite the aforementioned biochemical and morphological differences. This finding is corroborated by measurements of AFDW of the aggregated samples (see Table 2). It is noteworthy, however, that the HL×HS culture deviates somewhat from the general trend observed in the other culture conditions. Specifically, *D. viridis* cultured under these conditions shows generally higher near IR attenuation per unit biomass, likely linked to increased pigment absorptions in this spectral region. Comparison of these data to calculations of the Mie scattering profile of a simplified microalgae model (Quirantes and Bernard 2004) based on the observed variations in the effective cell diameters predicts a 180–240 % increase in the near IR scattering profile over a refractive index difference range of 0.06–0.26. Comparison of these calculated changes with respect to the experimentally observed ∼300 % increase in near IR attenuation suggests that diffuse scatter is responsible for ∼60–80 % of the observed optical attenuation at 800 nm.

**Table 2:** Summary of the measured parameters obtained in assessment of the photo-osmotic adaptation matrix. Error values correspond to 1−*σ* standard deviations for each of the measurements. For AFDW and the bioproduct yields, the errors originate from three replicate measurements from biomass that was pooled from the 20 semi-continuous harvesting intervals. For the remaining parameters, *n* = 20 per condition

	HL×HS	HL×MS	HL×LS	LL×HS	LL×MS	LL×LS
Biomass yield (day^−1^)						
Vcell (μm^3^)	252 ± 72	280 ± 89	696 ± 132	366 ± 111	335 ± 106	414 ± 125
Ncells (×10^3^ mL^−1^)	1961 ± 29	2982 ± 37	370 ± 24	1137 ± 26	1345 ± 33	173 ± 27
Biovolume (×10^−4^ mL^−1^)	4.94 ± 0.28	8.37 ± 0.33	2.58 ± 0.25	4.17 ± 0.33	4.52 ± 0.34	1.71 ± 0.46
Ash-free dry weight (g/L)	0.143 ± 0.007	0.218 ± 0.009	0.062 ± 0.006	0.117 ± 0.009	0.118 ± 0.009	0.04 ± 0.01
Bioproduct yield (fraction of dry weight)						
Carotenoids	0.044 ± .006	0.034 ± 0.006	0.032 ± 0.007	0.037 ± 0.005	0.029 ± 0.004	0.026 ± 0.005
Chlorophylls	0.016 ± 0.005	0.023 ± 0.004	0.023 ± 0.006	0.034 ± 0.006	0.031 ± 0.005	0.035 ± 0.006
Lipids	0.11 ± 0.03	0.09 ± 0.03	0.23 ± 0.05	0.11 ± 0.03	0.10 ± 0.03	0.26 ± 0.03
Carbohydrates	0.31 ± 0.04	0.34 ± 0.05	0.55 ± 0.06	0.27 ± 0.05	0.19 ± 0.04	0.46 ± 0.05
Proteins	0.14 ± 0.04	0.16 ± 0.04	0.29 ± 0.06	0.23 ± 0.04	0.26 ± 0.04	0.24 ± 0.04
Glycerol	0.56 ± 0.05	0.49 ± 0.05	0.08 ± 0.04	0.49 ± 0.05	0.52 ± 0.06	0.06 ± 0.05
Physiological biomarkers						
Cell granularity^a^	1.55 ± 0.14	1.63 ± 0.18	2.78 ± 0.22	1.02 ± 0.09	1.13 ± 0.10	2.23 ± 0.18
Debris content	0.57 ± 0.06	0.92 ± 0.14	0.72 ± 0.08	0.31 ± 0.19	0.96 ± 0.11	0.63 ± 0.10
Motility	0.90 ± 0.04	0.94 ± 0.05	0.82 ± 0.06	0.89 ± 0.03	0.91 ± 0.03	0.84 ± 0.05
Int. pH	7.63 ± 0.14	6.67 ± 0.10	6.78 ± 0.22	7.35 ± 0.14	6.98 ± 0.09	7.15 ± 0.20
Ext. pH	9.20 ± 0.04	9.27 ± 0.05	9.11 ± 0.06	9.19 ± 0.04	9.06 ± 0.06	8.82 ± 0.08
Int. Ca^2+ a^	3.73 ± 0.28	1.64 ± 0.26	3.2 ± 0.31	3.81 ± 0.26	2.33 ± 0.22	3.57 ± 0.30
Δ[NO_3_] (mM)	1.45 ± 0.12	1.08 ± 0.14	0.937 ± 0.19	0.741 ± 0.14	0.788 ± 0.09	0.821 ± 0.11
ΔNAD(P)H^a^	0.483 ± 0.044	0.73 ± 0.050	0.44 ± 0.052	0.35 ± 0.71	0.367 ± 0.043	0.567 ± 0.055
Photosystem II parameters						
Max yield, *Y* ^max^(II)	0.69 ± 0.03	0.64 ± 0.02	0.59 ± 0.05	0.72 ± 0.03	0.69 ± 0.03	0.65 ± 0.04
Low PAR yield, *Y*(II)	0.47 ± 0.03	0.39 ± 0.03	0.28 ± 0.04	0.53 ± 0.03	0.47 ± 0.02	0.34 ± 0.04
Low PAR *Y*(NPQ)/*Y*(NO)	0.47 ± 0.21	0.72 ± 0.19	1.48 ± 0.23	0.21 ± 0.13	0.43 ± 0.18	0.56 ± 0.12
High PAR yield, *Y*(II)	0.24 ± 0.04	0.17 ± 0.02	0.14 ± 0.04	0.27 ± 0.03	0.24 ± 0.02	0.18 ± 0.03
High PAR *Y*(NPQ)/*Y*(NO)	1.96 ± 0.23	1.90 ± 0.25	1.00 ± 0.20	0.51 ± 0.27	1.11 ± 0.21	1.00 ± 0.23

^a^Observables whose relative intensities are obtained spectrophotometrically

**Fig. 3:**
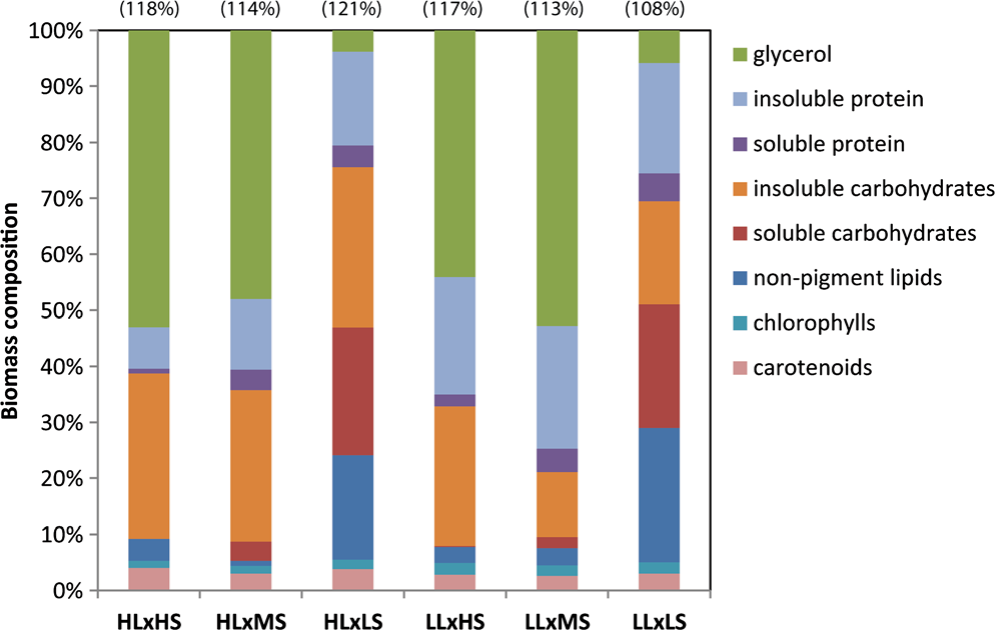
Mass balance of the dry weight of semi-continuous cultures of *D. viridis* in each of the photo-osmotic conditions. Overestimation of the AFDW mass balance for the pooled biomass samples (*n* = 20 per condition) are depicted in *parenthesis* above the results obtained for each culture condition

The compositional mass balance of the major biochemical pools of the biomass cultivated in the various photo-osmotic regimes are depicted in Fig. 3. The most significant compositional variations were observed in the quantities of glycerol, followed by soluble carbohydrates and (non-pigment) lipids. For each of the observed compositional shifts, the dominating environmental variable was change in salinity, with the largest effects predicated by shifts to or from low (marine) salinity levels. The total glycerol corresponded to 53–47 % for high salt (∼2.36 M NaCl), 52–47 % for medium salt (∼1.36 M), and 4–6 % for marine medium. The soluble carbohydrates corresponded to <1 % for high salt, 3–5 % for medium salt, and 22–24 % for marine medium. The non-pigment lipid fraction corresponded to 3–5 % for high salt, 2–5 % for medium salt, and 18–23 % for marine medium. The most dramatic shifts that were dominated by light intensity levels were insoluble protein and insoluble carbohydrate, although substantial changes were also observed for chlorophyll (*a*+*b*) and carotenoids, despite their low abundance relative to the other fractions. The insoluble protein fraction corresponded to 8–15 % for high PAR and 14–18 % for low PAR. The insoluble carbohydrate fraction corresponded to 28–30 % for high PAR and 11–21 % for low PAR. For each assessment of compositional mass balance, the summed mass fractions exceeded the mass of the biomass between 6–22 %, indicative of some combination residual salts and cross-talk (additive noise) among the various measurements. Based on additive accumulation of error distributed among the various biochemical fractions, the approximate error for each fraction is <3 %. For completeness, the overestimates relative to the measured dry weight of the biomass are stated in parenthesis above the data for each culture condition.

**Fig. 4:**
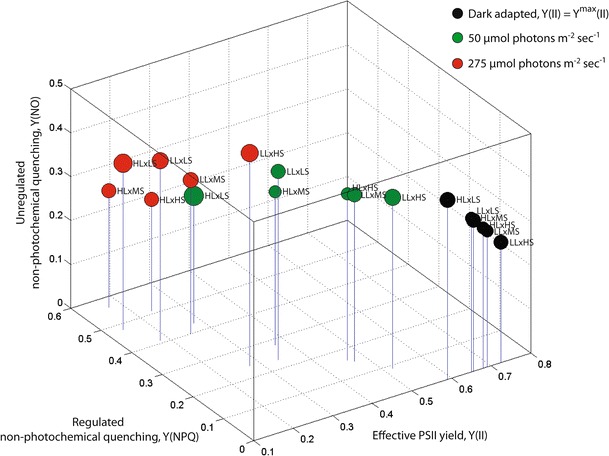
PSII parameters obtained for each of the culture conditions, including maximum PSII yield, *Y* ^max^(II); effective PSII yield, *Y*(II); regulated non-photochemical quenching, *Y*(NPQ); and background or unregulated non-photochemical quenching, *Y*(NO). Data from dark adapted cultures are represented with *black markers*, low PAR (50 μmol photons m^−2^ sec^−1^) illuminated samples with *green markers*, and high PAR (275 μmol photons m^−2^ sec^−1^) illuminated samples with *red markers*. The marker sizes correspond to the 1−σ standard deviation (*n* = 20) obtained from the full data collection period

Photosynthesis parameters, including the maximum [*Y* ^max^(II)] and effective [*Y*(II)] quantum yields of PSII, and regulated (photo-protective) non-photochemical [*Y*(NPQ)] and unregulated non-photochemical quenching [*Y*(NO)] obtained from RLC excitation modulated fluorescence experiments are depicted in Fig. 4. The results are summarized for dark adaptation, at the 50 μmol photons m^−2^ sec^−1^ exposure—corresponding to the low light growth regime, and at the 275 μmol photons m^−2^ sec^−1^—corresponding to the high light growth regime, respectively. For each data set, clear trends are evident and related to the specified growth condition, with the dominant effects dictated by changes in salinity. For the dark adapted state, *Y* ^max^(II) increased with elevated salinity and reduced light exposure from 59 % (HL×LS) to 73 % (LL×HS). Culturing at low light intensities consistently increased the maximum photosynthetic yield by ∼5 % compared to the corresponding high light cultures. At the low light exposure during the RLC, *Y*(NPQ) is significantly elevated relative to unregulated processes *Y*(NO) for cultures grown at reduced salinities and increased PAR. At the high light exposure during the RLC, the salinity dependence of *Y*(NPQ) relative to *Y*(NO) is dramatically reduced, although *Y*(NPQ) continues to be elevated by 1.2–2× for cultures grown at increased PAR. The highest *Y* ^max^(II) and *Y*(II) values were consistently observed in the low light, high salinity cultures, indicating enhanced overall photosynthetic productivity under the elevated salinity regimes.

In addition to the biomass yield, biochemical composition, and photosynthetic parameters, a number of physiological biomarkers were measured across the photo-osmotic adaptation matrix for correlation with the materials and productivity data. These biomarkers included cellular granularity, content of cellular debris, cellular motility, intracellular and extracellular pH, nitrate consumption, intracellular calcium, and turnover of NAD(P)^+^/NAD(P)H. With the exception of extracellular pH and nitrate consumption, rigorous quantitation of the biomarkers was not intended; instead the goal was to provide relative metrics for gaining a deeper understanding of intercellular signaling and energetics underlying the biochemical and morphological properties of the algae. Among the conditions tested cellular granularity exhibited the following trend: HL×LS > LL×LS > HL×MS > HL×HS > LL×MS > LL×HS; the relative abundance of cellular debris exhibited the following trend: HL×MS > LL×MS > HL×LS > LL×LS > HL×HS > LL×HS; the relative cellular motility exhibited the following trend: HL×MS > LL×MS > LL×HS > HL×HS > LL×LS > HL×LS; the intracellular pH exhibited the following trend: HL×HS > LL×HS > LL×LS > LL×MS > HL×LS > HL×MS; the extracellular pH exhibited the following trend: HL×MS > HL×HS > LL×HS > HL×LS > LL×MS > LL×LS; the nitrate consumption exhibited the following trend: HL×HS > HL×MS > HL×LS > LL×LS > LL×MS > LL×HS; the intracellular calcium exhibited the following trend: LL×HS > HL×HS > LL×LS > HL×LS > LL×MS > HL×MS; and the NAD(P)^+^/NAD(P)H turnover during saturation pulse illumination exhibited the following trend: HL×MS > LL×LS > HL×HS > HL×LS > LL×MS > LL×HS.

**Fig. 5:**
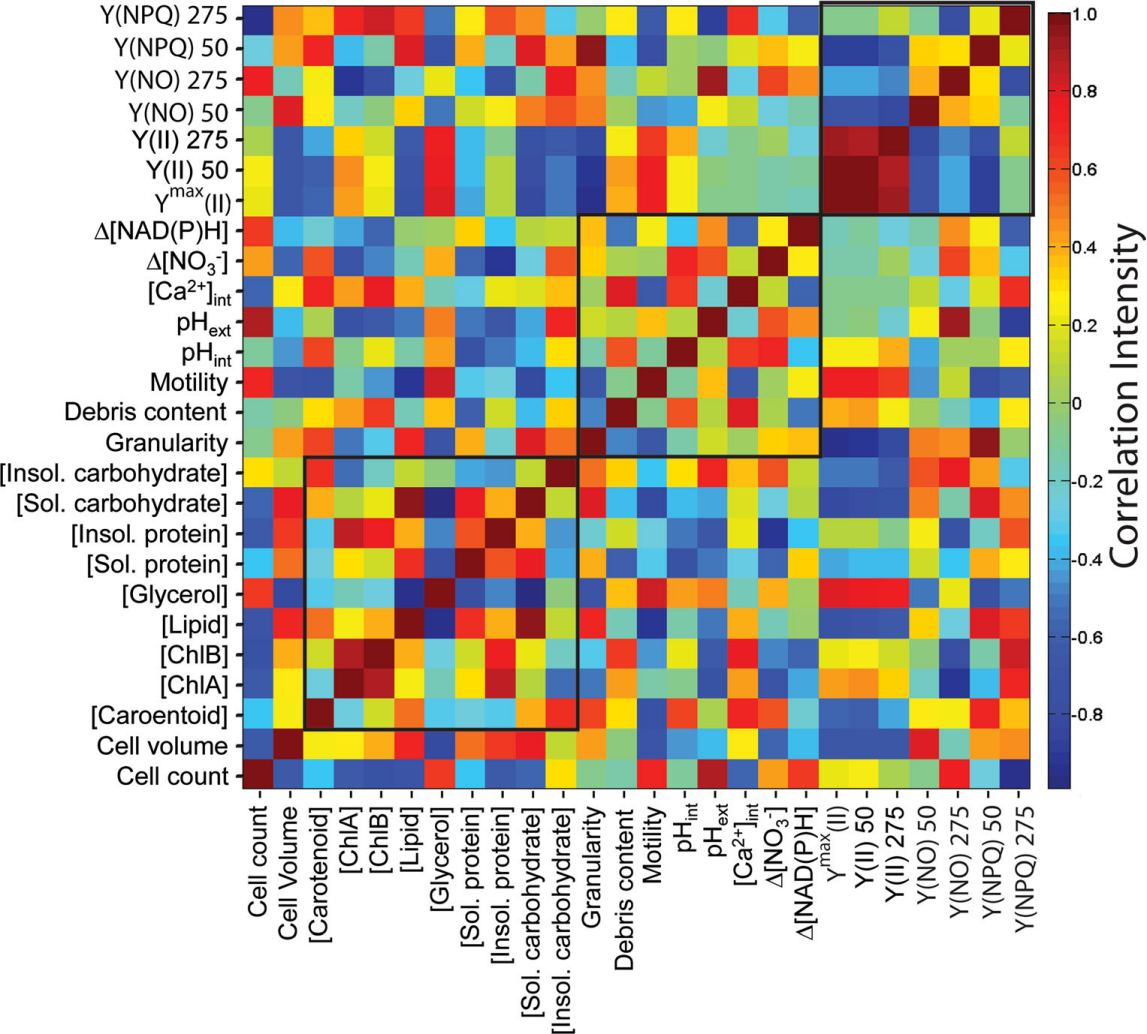
Correlation matrix for each of the measured observables for the photo-osmotically adapted *D. viridis* cultures. *Black boxes* indicate groupings of related parameters, i.e., along the diagonal from *lower left* to *upper right*: bioproduct yields, biomarker intensities, and PSII parameter yields. Data points reflected across the *lower left* to *upper right* diagonal are redundant

In order to succinctly summarize the results, a correlation matrix of the various observables were constructed based on the photo-osmotic conditions, and is depicted in Fig. 5. Although a wide variety of measurements were performed, normalization of the results by ${P}_i=\frac{O_i}{{\displaystyle \sum {O}_i}}-\left\langle \frac{O_i}{{\displaystyle \sum {O}_i}}\right\rangle$, where *O*_*i*_ is the parameter value for the *i*th environmental condition, can be shown to preserve the original variance of data and support analyses which are independent of sensitivity variations among the various assays, if the measurements can be shown to be within acceptable detection limits. Once scaled to unit variance, the data were then constructed into a covariance matrix and subsequently into a correlation matrix using built-in functions in Matlab. In the figure, the various parameters are grouped by category from left to right and bottom to top: biomass quantity, biochemical composition, physiological biomarkers, and photosynthetic parameters, respectively. Correlations among members of the same category are boxed along the diagonal of the matrix, and data points reflected across the diagonal are redundant. Noteworthy among the positively correlated observables are increased concentrations of soluble carbohydrates and total lipids, increased low PAR photo-damage and cellular granularity, increased photochemical quenching and extracellular pH, and increased concentration of chlorophylls and structural proteins. Noteworthy among the observables with highly negative correlation coefficients (less than −0.8) are the relative concentrations of soluble carbohydrates and glycerol, increased (maximum and low PAR) photosynthetic yield and high cellular granularity, cellular volume and the relative concentration of glycerol, and the relative concentrations of glycerol and total lipids. Analysis of the principle components of the results matrix was also performed to identify and rank variations not predicated by the environmental conditions tested. According to Hotelling’s *t* ^2^ values generated by this principle components analysis, variations in the cellular motility and the concentration of cellular debris were least dependent on the photo-osmotic conditions investigated. Variations in these parameters may instead have resulted from temporal effects over the course of the long-term data collection not specifically treated in this analysis.

**Fig. 6:**
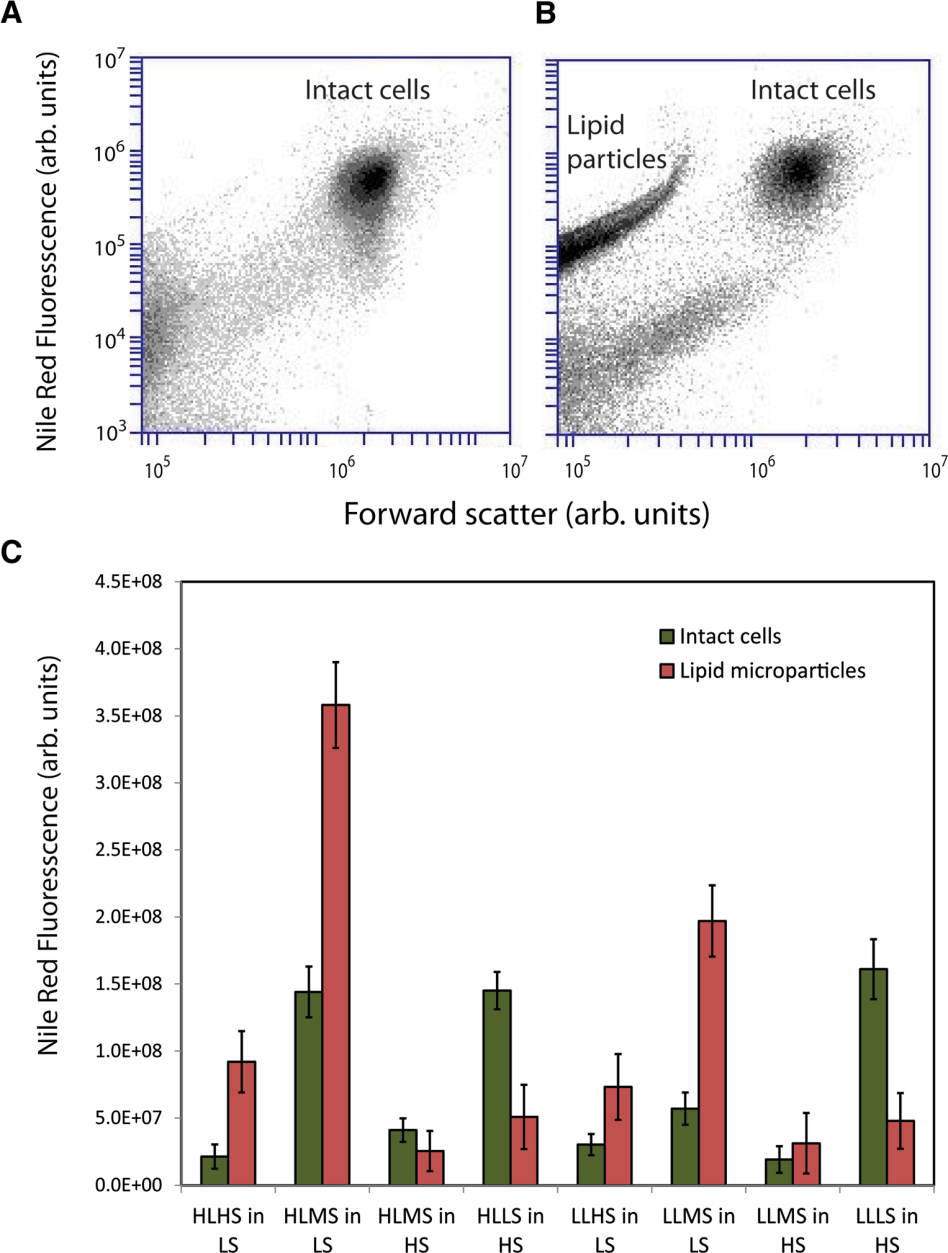
Lipid microparticle release under osmotic drift. **a** Flow cytometry scatter plot for total lipid (as indicated by Nile Red fluorescence intensity) versus particle diameter (as indicated by forward scatter) for the first day of osmotic shock in the HL×MS culture. **b** Repeat scatter plot for the fourth day of osmotic shock, corresponding to the second harvesting increment. **c** Summary of the total lipid content in the intact cell and lipid microparticles as indicated by Nile Red fluorescence, error bars indicate 1 − *σ* standard deviations (*n* = 3)

Following the 6-week semi-continuous culture and harvesting of *D. viridis* in the specified photo-osmotic culture conditions, each culture was subject to continued semi-continuous culture in either of the extreme of salinity (HS or LS) for additional harvesting cycles (8 days). The most noteworthy observation was made by comparing flow cytometry data of Nile Red stained cells at day 0, corresponding to the initiation of the dynamic osmotic adaptation regimen, to replicate experiments performed at the second harvest interval of the osmotic shock treatment (4 days after initiation of the regimen). Figure 6a, b depicts scatter plots corresponding to effective particle diameter (FSC) and Nile Red intensity (FL2) for days 0 and 4, respectively. These data clearly reveal a band of highly lipophilic microparticles ranging in diameter from ∼1–3 μm in addition to the expected grouping of intact *D. viridis* cells. Comparison of Nile Red intensities per unit volume from each population suggests that the lipid concentration in the microparticles is up to ∼1,275 % that of the algal cells. Figure 6c depicts the total lipid mass balance (as indicated by Nile Red intensity) of the lipophilic microparticles compared to the remaining intact cells. In each case of cells semi-continuously cultured in decreasing salinity media, the total lipid was highly concentrated in the lipophilic microparticle phase, whereas in the cases of cells resuspended in increased salinity, the total lipid was primarily concentrated in the algae. The highest overall lipid was observed in MS cultures diluted by marine (LS) medium; however, it is noteworthy that the high PAR culture (HL×MS) exceeded the low PAR culture (LL×MS) for total lipid by a factor of ∼200 %. Because other debris and instrumental background dominate the flow cytometric signal for particles less than ∼1 μm in diameter, the contribution to the total lipid and the ratio of the lipid in the microparticles versus the intact cells should be treated as the lower bound of the estimates.

## Discussion

Continuous or semi-continuous culturing of algae biomass provides the opportunity to increase yield by eliminating lag and other effects that occur in conventional batch cultivation. In this work, we sought to identify environmental factors that would allow selective harvesting of lipids from an algal culture as well as high log-phase biomass growth yield at semi-continuous intervals. Salinity and solar radiation are ubiquitous but highly variable factors that impact the yield and composition of algae biomass in outdoor cultivation. Therefore, we investigated the potential for *D. viridis*—a species with high tolerance to a wide range of environmental conditions and a history of successful outdoor cultivation—as a candidate for combining semi-continuous biomass production with selective release of bioproducts (milking), a process which has only been previously achieved by cell lysis with chemical solvents (Hejazi et al. 2004; Kleinegris et al. 2010a). A combination of single-cell imaging, bulk spectrophotometry, and cell counting were used to establish a simple near IR attenuation-based method for quantitative harvesting of the log-phase growth across each of the photo-osmotic conditions. Despite substantial differences in morphology and composition (resulting in ∼300 % variance in the single-cell optical attenuation profiles across the various culture conditions), the 800 nm optical density was found to correlate linearly with biomass accumulation. A similar approach was used previously for assessment of high-density outdoor algal cultures (Gitelson et al. 1995), however the authors of that study suggested that further investigation was necessary to determine if their approach could be generalized to algae biomass of varying composition. The method described here provides accurate quantification of biomass in samples of highly variable biochemical composition, cell physiology, nutrient concentrations, salinity, and light intensity—parameters known to cause substantial errors in pigment-based spectrophotometric approaches such as chlorophyll *a* absorbance (Meeks 1974; Riemann et al. 1989). Because of the unreliability of pigment-based spectroscopic indicators of biomass productivity, other investigators have employed NAD(P)H fluorescence as an indicator of photosynthetic productivity in marine microalga (Steigenberger et al. 2004). In this study, however, we found that the correlation between NAD(P)H and biomass productivity was ambiguous (<0.5) when cell number and cell size parameters were considered simultaneously, likely related to metabolic responses involved in haloadaption that will be discussed in more detail in a subsequent paragraph. We therefore propose that the spectroscopic method described here could provide improvements in online measurement of algae cultures for increasing overall yields by crop management, e.g., for determination of optimal cultivation parameters and harvesting intervals (Havlik et al. 2013). In the studies describe here, semi-continuous cultures that were established using the near IR attenuation-based harvesting scheme were found to be stable over the course of the 6-week study, however the biomass yields that were obtained varied substantially across the different culture conditions. Specifically, the maximum yield was observed in the HL×MS culture, followed by the HL×HS, LL×MS, LL×HS, HL×LS, and LL×LS. The resulting biomass yield parameters, biochemical mass balance, and morphological, photosynthetic, and biomarker parameters are summarized in Table 2. It should also be noted that in addition to morphological details, hyperspectral confocal fluorescence imaging with multivariate analysis provided a set of spectral components composed of fluorescence and resonance Raman emission for unambiguous identification of intracellular carotenoid and chloroplast bodies in *Dunaliella*. These findings supplement previously described fluorescence imaging and Raman spectroscopic methods (Collins et al. 2011; Kleinegris et al. 2010b; Vermaas et al. 2008) for label-free quantitation of carotenoid production, a well-established high-value product of *Dunaliella*.

Photosynthetic parameters and a number of biomarkers related to photo-osmotic adaption and carbon metabolism were measured for purposes of classification and relation of the bioproduct yields to fundamental cellular processes. Correlation and principal component analyses revealed distinct patterns of interrelationship among the variables, providing a set of biological bounds for multi-objective and multi-modal biomaterials optimization for *D. viridis*. Since the correlation matrix includes data from each harvest increment over the 6-week assay, the indicated correlation values should be statistically robust. Furthermore, this approach provided a subset of overlapping observations for registration of the bulk and imaging results, correction of osmotic perturbations to optical measurements of the culture, and identification of measured parameters that are most and least dependent on the PAR intensities and salinity levels explored in this study. Together, the data presented here reinforce previous observations that the species under study is in fact *D. viridis* (Borowitzka and Siva 2007)—as opposed to *D. salina*, as reported by the Culture Collection of Algae at University of Texas—based on the moderate halophilicity indicated by the phenotypic parameters. Furthermore, recent taxonomic analysis of the genus *Dunaliella* indicates that such misidentification of strains among the culture repositories is likely wide-spread (Assunção et al. 2013).

A previously well-documented and dominant feature of the global correlation analysis presented here is the dynamic interconversion (anti-correlation) between glycerol and soluble carbohydrates (starch) in response to varying salinity. Previous research indicates a four-step glycerol cycle in *Dunaliella* (Wegmann 1979). According to enzymatic assays, glycerol synthesis is initiated by reversible conversion of dihydroxyacetone phosphate to glycerol-3-phosphate catalyzed by glycerol-3-phosphate dehydrogenase (Haus and Wegmann 1984a, b). This is followed by an irreversible dephosphorylation by glycerol-3-phosphatase (Sussman and Avron 1981). Conversion of glycerol back to dihydroxyacetone phosphate proceeds by reversible oxidation to dihydroxyacetone by the NADP^+^-dependent glycerol dehydrogenase (Ben-Amotz and Avron 1974) and subsequent irreversible phosphorylation catalyzed by dihydroxyacetone kinase (Lerner et al. 1980). According to this cycle, dihydroxyacetone phosphate is the key intermediate of cyclic glycerol metabolism in *Dunaliella*; the data depicted here suggest that the dominant source of this intermediate originates from starch breakdown and is largely independent of variable photon flux density and PSII parameters. The intracellular concentration of multiple ions (e.g., H^+^, Ca^2+^, Na^+^, K^+^) is likely involved in the regulation of glycerol metabolism. Data from the photo-osmotic conditions tested here suggest that constitutively elevated intracellular pH, but not intracellular Ca^2+^, is tightly linked to glycerol concentration, likely by modulating the function of the enzymes involved in the system (Busa and Nuticelli 1984). However, previous observations of transient ion fluxes (esp. Na^+^ and K^+^) implicated in hypertonic adaptation (Ehrenfeld and Cousin 1984; Ginzburg 1981) suggest that constitutive elevation of such ions is likely not be required for long-term elevation of intracellular glycerol. Interestingly, interconversions of similar magnitude of that between glycerol and starch are also evident for glycerol and soluble proteins and lipids in the photo-osmotic variants explored here. Thus, it is likely that multiple metabolic pathways are initiated to provide the dihydroxyacetone intermediate for glycerol production. Furthermore, the high overall correlation between glycerol production and maximum and effective photosynthetic yield parameters suggests that the compound not only provides for regulated osmotic balancing, but is dominant factor for determining the total yield and biochemical composition of the biomass under variable environmental conditions. Although glycerol is typically viewed as a low-value byproduct of biofuels production (e.g., from biodiesel transesterification), because of the high degree of carbon reduction in this compound there is potential to produce fuels and other reduced chemical intermediates using biochemical conversion approaches such as anaerobic fermentation (Clomburg and Gonzalez 2013). For conditions that proved most optimal for high growth rate, high correlation intensities were observed between the photosynthetic yield parameters and nitrogen consumption. The fact that the correlation between these two parameters in the global analysis is ambiguous provides evidence for compensatory mechanisms for maintaining growth under suboptimal light/salinity regimes by a relative increase in nitrate uptake per unit biomass. This result is consistent with a similar effect that was reported previously in *D. tertiolecta* in studies of simultaneous light and nitrate limitation (Sciandra et al. 1997).

Finally, release of lipid microparticles from the algae cells was observed during a variety of photo-osmotic drift conditions. Assessment of the mass balance of the lipid fraction retained in the cells and released into the media (Fig. 6c) suggest that a process that combines a moderately high salinity cultivation medium (∼1.36 M NaCl) and high light intensities with intermittent induction of hypotonic drift (or a biochemically simulated version thereof) would provide high overall lipid yield and result in release of ∼62 % of the accumulated lipids into the culture medium. Analysis of the cell counts from the initial and final days of this assay indicate that significant growth yield loss (∼28 %) is incurred as a result of the shifted salinity. However, the fact that >60 % of the lipid is transferred to the bulk medium suggests that the mechanism for the observed lipid release is not entirely dominated by lethal or growth-arresting cell lysis, but that reversible permeation may also play a significant role. Although a precedent for osmotically induced milking of bacteria has been demonstrated previously (Sauer and Galinski 1998), to our knowledge this is the first report showing potential for similar techniques for isolation of microalgal bioproducts. Combining this strategy with semi-continuous biomass harvest could likely be achieved using microparticle-selective filtration, lipid adsorbents, or other passive approaches and therefore prevent wholesale lysis of the biomass by acute osmotic changes (Ginzburg et al. 1990) or direct and interfacial exposure to chemical solvents reported previously (Kleinegris et al. 2011). The energy savings potential provided by avoidance of dewatering the biomass using such a process could account for as much as a 30 % reduction in the total cost of bioproducts and fuels from microalgae (Greenwell et al. 2010). Furthermore, the process should be amenable for isolation of lipophilic high-value products (such as carotenoids) prior to hydrothermal upgrading of wet biomass slurries to fuels (Elliot et al. 2013).
